# Circumscribed Congenital Alopecias Harbouring Dual Lesions

**DOI:** 10.4103/0974-2077.69025

**Published:** 2010

**Authors:** Shalinee Rao, Amutha Janaki, D Kamakshi, V Srinivasan

**Affiliations:** *Department of Pathology, Sri Ramachandra University, Porur, Chennai-600 116, India*; 1 *Department of Plastic Surgery, Sri Ramachandra University, Porur, Chennai-600 116, India*

**Keywords:** Alopecia, dermatophytosis, nevus sebaceous, scalp, syringocystadenoma papilliferum

## Abstract

Treatment of alopecia is often challenging for the clinician as it includes a spectrum of lesions ranging from congenital to acquired causes. We present three cases of congenital circumscribed alopecia, present since birth, clinically diagnosed as nevus sebaceous. Histopathological examination of the excised tissue showed syringocystadenoma papilliferum with dermatophytosis in one and nevus sebaceous with dermatophytosis in the other two cases. Although complete excision is the treatment of choice for these lesions, an antifungal agent is needed to eradicate the concurrent superficial mycosis. A careful histopathological examination of the lesional skin helps in identifying such unexpected dual lesions that would need further treatment.

## INTRODUCTION

Alopecia is the pathological loss of hair, which could be generalised or localised. The clinical manifestation, age of patient at the time of presentation and presence of associated symptoms are the vital information needed to clinically differentiate congenital and acquired diseases.[1] Alopecia is histologically categorized as scarring or non-scarring depending on whether or not the hair follicles are destroyed.[2] It is important to identify the underlying cause of alopecia, as therapeutic options vary with aetiology.[2] The psychosocial effect and cosmetic importance further necessitate treatment of alopecia. We present three cases of childhood circumscribed alopecia of different aetiology, all harbouring a common acquired lesion.

## CASE REPORTS

### Case 1

**Figure 1:**
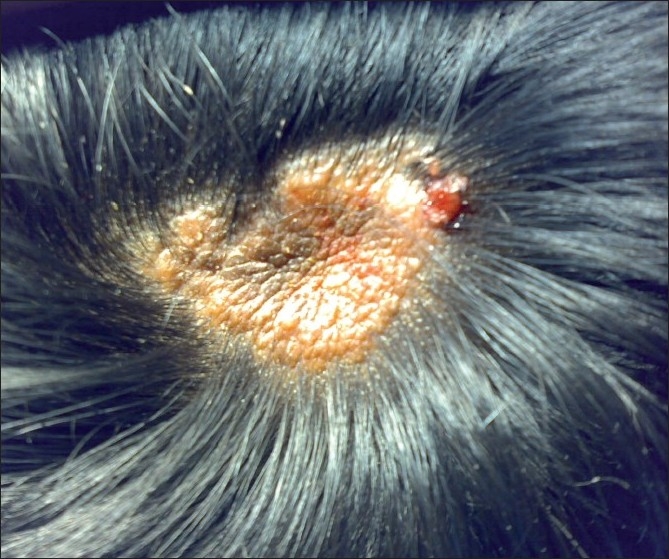
A circumscibed area of alopecia on the vertex of scalp covered with yellowish exudate

A nine-year-old boy was brought with complaints of localised baldness of his scalp, since birth. The surface area of baldness remained constant with no increase or decrease in size, since birth. However, this became greasy with intermittent ulceration. The patient was treated a few days earlier by a dermatologist, who prescribed the local application of antibiotics. There was no abnormality detected on general physical examination and systemic examination. Local examination of the scalp revealed an irregular circumscribed hairless patch, 3 × 3 cm over the vertex of the scalp, with yellowish surface exudates [Figure 1]. A clinical diagnosis of nevus sebaceous was considered and excision of the patch was performed.

**Figure 2:**
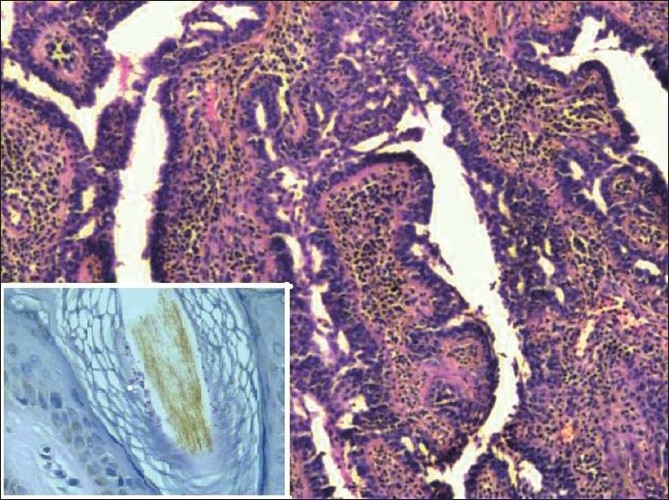
Skin with adnexal tumour syringocystadenoma papilliferum, showing abundant plasma cells in the stroma (H and E ×40); inset shows fungal spores within the hair follicle (Periodic acid Schiff ×100)

Microscopic examination of the processed tissue showed a skin adnexal tumour with invagination of its epithelium into the dermis [Figure 2]. The tumour was lined by squamous epithelium on the surface and the invaginated portion was lined by a double layer of cells consisting of cuboidal and columnar epithelium. Many plasma cells were seen in the stroma of the tumour. The rest of the skin showed normal sebaceous glands with mature hair follicles. In addition, the stratum corneum and few hair follicles showed spores of fungal organism in the skin adjacent to the tumour [Figure 2]. The dermis showed a non-specific chronic inflammatory cell infiltrate with a focus of foreign body giant cell reaction. Periodic acid Schiff stain (PAS) with diastase and Gomori’s methnamine silver (GMS) [Figure 2 inset] stains confirmed the presence of dermatophytes. Syringocystadenoma papilliferum with dermatophytosis of the scalp was the final histopathological diagnosis.

### Case 2

A 12-year-old boy was brought with a history of a localised area of baldness over the scalp since birth, which did not alter in size. However, occasional bleeding was noticed from this lesion just after combing.

**Figure 3:**
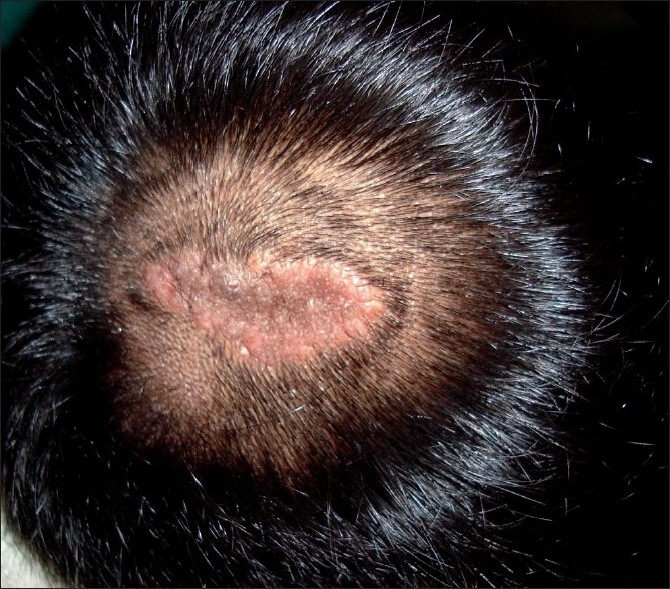
Scalp with an irregular patch of hair loss showing mild nodularity

There was no abnormality detected on general physical examination and systemic examination. Local examination of the scalp revealed a circumscribed alopecic patch, measuring 3 × 1.7cm, localised to the vertex of the scalp, with slight nodularity [Figure 3]. However, there was no discharge, ulcer or scarring in the lesion. A clinical diagnosis of nevus sebaceous was considered and the lesion was excised.

**Figure 4:**
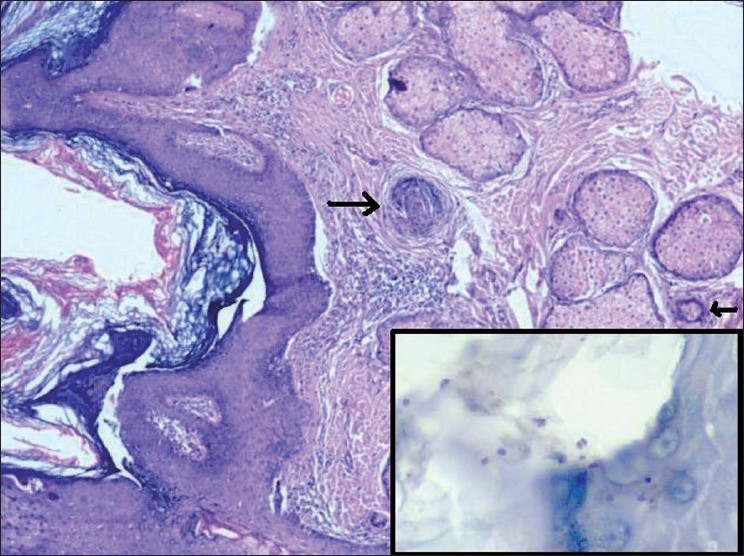
Skin shows irregular acanthosis, papillomatosis and hyperkeratosis of the epidermis with immature hair follicles (arrow) and hypertrophied sebaceous glands (H and E ×40); inset shows occasional polymorph and many fungal spores in the stratum corneum (Periodic Schiff stain ×200)

Histopathological examination showed skin with irregular acanthosis, papillomatosis and hyperkeratosis [Figure 4]. The dermis showed abundant sebaceous glands and immature hair follicles, few with infundibular dilatation [Figure 4]. Occasional apocrine glands and foci of chronic inflammatory cell infiltrates were also seen. Stratum corneum and hair follicles showed fungal spores confirmed by PAS with a diastase stain [Figure 4 inset] and Grocott’s silver (GMS) stain. The final opinion on the biopsied specimen was nevus sebaceous with dermatophytosis.

### Case 3

A 22-year-old lady presented with complaints of hair loss over the scalp, since birth, with no increase in size. She also gave a history of itching for the past two months.

No abnormality was detected on general physical examination or systemic examination. On local examination a linear patch of alopecia was noticed over the occipital region measuring 3 × 1 cm. There was no evidence of surface ulceration or exudation. An excision of the lesion was performed and sent for histopathological examination.

The excised scalp tissue showed focal acanthosis, papillomatosis and hyperkeratosis. The dermis showed prominent sebaceous glands, undifferentiated hair follicles, few with infundibular dilatation and non-specific chronic inflammatory cell infiltrate. The stratum corneum and hair follicles showed few polymorphs and fungal spores which were confirmed by PAS with diastase and GMS stains. A final diagnosis of nevus sebaceous with dermatophytosis was given.

## DISCUSSION

In our first case, the alopecia was due to syringocystadenoma papilliferum – a rare sweat gland tumour that commonly presents as a hairless plaque or as papules on the scalp. It occurs usually in the head and neck region, but may also be located in the trunk or extremities. The lesion occurs during early childhood, and in half the cases, presents at birth and increases in size at puberty, becoming papillomatous and at times everted.[3] Syringocystadenoma papilliferum usually develops within a nevus sebaceous, but may also, occur *de novo*.[4] The absence of ectopic pilosebaceous structures on histopathology favoured the latter possibility in the first case. The other two cases also presented with alopecia since birth, which were clinically and histopathologically diagnosed as nevus sebaceous. Nevus sebaceous is a rare congenital hamartoma, otherwise known as organoid nevus, consisting of a malformed sebaceous gland, the epidermis and other appendageal structures. It occurs more commonly on the scalp and face, but can also be found in the neck, trunk and extremities.[5] In the scalp, it initially presents at birth or early childhood as a circumscribed, slightly raised, hairless plaque that may be linear, round, or irregular, and becomes verrucous or nodular at puberty.

The unique finding in these three cases was concurrent dermatophytosis with folliculitis, which was not suspected clinically. Tinea capitis is an infection of the scalp and hair shaft caused by dermatophytic fungi. Clinical diagnosis of tinea capitis can be challenging as symptoms can vary from minimal pruritus with no hair loss to severe tenderness, purulence and alopecia with / without permanent scarring.[6]

Dermatophytosis is primarily a clinical diagnosis that is performed with the aid of Wood’s light examination or direct microscopy of a skin scrapping with potassium hydroxide, and only rarely does it require a skin biopsy for confirmation.[7] Microscopically the tissue changes include psoriasiform and spongiotic epidermis with a vesiculobulous to granulomatous reaction. Altered keratinisation and sandwich sign (neutrophils entrapped between keratin layers) in the epidermis are other soft signs to suspect fungal infections. All our cases showed focal acanthosis and non-specific, chronic inflammatory reaction in the upper dermis with giant cell reaction in one. The fungus uses the non-keratin layers of the epidermis for nutrition and keratin for protection from the host response.[7] Fungi causing tinea capitis belong to the trichophyton and microsporum species, which exist either in the hyphael forms with branching and septations or as spores. In our cases, the fungal spores were noted in the stratum corneum and were also seen involving the hair follicles. We confirmed the presence of fungal spores by utilising special stains, PAS with diastase and GMS. The addition of enzyme diastase in PAS staining removes glycogen granules in the epidermal cells, which may otherwise give false positive results as they may resemble fungal spores.

Syringocystadenoma papilliferum requires a simple excision with no further treatment. Nevus sebaceous is treated either by dermabrasion, dermablation, or surgical excision. An excision biopsy in early childhood is preferred for this lesion as it can give rise to neoplasms, some of which may be malignant and not always would there be clinical signs of malignancy.[5] Fungal infection may coexist with another inflammatory or neoplastic lesion of the skin.[7] The acquired lesion could be missed if the clinical features are not very evident, or undue to attention being given only to the primary lesion, which had brought the patient to the clinician. Features of superficial mycosis were not suspected or clinically evident in all our cases. Treatment of syringocystadenoma papilliferum and nevus sebaceous would be complete with excision of the entire lesion, but the simultaneous presence of superficial mycoses needed further therapy with antifungal agents, for eradication of mycoses in all the three cases.

The presented cases highlight the fact that a careful histopathological examination of lesional skin could help in identifying unexpected lesions with dual aetiology, which may need further treatment. A meticulous examination of the tissue section by the histopathologist helps in identifying such unexpected concurrent lesions.
